# Construct Validity of the Mentalization Scale (MentS) Within a Mixed Psychiatric Sample

**DOI:** 10.3389/fpsyg.2021.608214

**Published:** 2021-05-28

**Authors:** Felix Richter, Dagmar Steinmair, Henriette Löffler-Stastka

**Affiliations:** ^1^Department of Psychoanalysis and Psychotherapy, Medical University of Vienna, Vienna, Austria; ^2^Karl Landsteiner University of Health Sciences, Krems, Austria; ^3^Department of Ophthalmology, Oculoplastics and Orbital Surgery, Karl Landsteiner University of Health Sciences, Sankt Pölten, Austria

**Keywords:** mentalization scale, mentalizing, reflective functioning scale, psychiatry, validity

## Abstract

**Introduction:** The concept of mentalizing is nowadays widely used in research as well as in clinical practice. Despite its popularity, the development of an economic assessment is still challenging. The Mentalization Scale appears to be a promising measurement with good psychometric properties but lacking convergent validity with the Reflective Functioning Scale.

**Objective:** This study aims to test the construct validity of the Mentalization Scale through correlations with the gold standard, the Reflective Functioning Scale, within a clinical sample. Furthermore, it was of interest to replicate its internal consistency.

**Methods:** Twenty-six inpatients of an acute psychiatric ward in Vienna were given the Mentalization Scale (MentS). They were interviewed with the Brief Reflective Function Interview, which was coded with the Reflective Functioning Scale. Correlations and internal consistency were calculated.

**Results:** Concerning the primary aim of this study, the validity was satisfactory for the MentS whole-scale mentalizing as well as for the subscales self- and other-oriented mentalizing. Internal consistency was lower to the findings of the developer and close to the 0.70 threshold.

**Conclusion:** Our findings could foster the psychometric properties of the MentS. Furthermore, the MentS seems to be a promising measurement tool for detecting different dimensions of reflective functioning. Limitations and further research are discussed.

## Introduction

Mentalizing is described as an imaginative ability to understand the behavior of others as well as oneself based on mental states like feelings, wishes, or beliefs to give meaningful interpretations on social interactions (Fonagy and Target, 1997). In its origin, the capacity to mentalize was conceptualized in a more cognitive-biological explanatory approach (Taubner, 2015) as the theory of mind (Premack and Woodruff, 1978). Fonagy and Target (Fonagy and Target, 1997) were elaborating with their conceptualization the importance of the interpersonal aspects of the development of the ability to mentalize and operationalize reflective functioning (RF).

Nowadays, the term “mentalizing” is widely used in clinical practice, often casually as a pre-diagnostic assessment (Luyten et al., 2019). A more detailed exploration of mentalizing capacity is sometimes difficult to integrate into the clinical routine due to the lack of economic measurements. The Reflective Functioning Scale (RFS; Fonagy et al., 1998) is regarded as the gold standard for the assessment of mentalization, but because of its complex analysis (i.e., interviewing, transcribing, and coding), it is very time-consuming. Likewise, in clinical practice, the research is confronted with the need for cost-efficient RF assessments to enable studies with bigger sample sizes and reach patients with more severe psychopathology (Fonagy et al., 2016). Beneath assessing the general RF, it is of growing interest to examine the different dimensions of RF. Various psychopathologies show specific deficits in these dimensions of RF (Choi-Kain and Gunderson, 2008; Luyten et al., 2019). In clinical practice as well as in research, it is of great importance to explore these specific deficits to foster therapeutic interventions. To date, self-report measurements of RF are limited. Oftentimes, similar constructs related to mentalization like empathy, mindfulness, or alexithymia are used to assess parts of mentalizing (Fonagy et al., 2016).

Fonagy et al. (2016) developed a short questionnaire, the Reflective Functioning Questionnaire (RFQ). The RFQ was theory driven and was constructed based on the concept of RF. Fonagy et al. (2016) highlighted that a self-report of mentalizing is facing the problem, that is, for stating the own capacity of oneself to mentalize, self-reflection itself is needed. Therefore, they focused on a specific aspect of RF, the certainty and uncertainty of mental states, which underlines the two subscales. Construct validity was satisfactory with similar constructs and was distinguished between healthy controls and borderline patients, which got replicated (Badoud et al., 2015; Fonagy et al., 2016; Morandotti et al., 2018).

Dimitrijević et al. (Müller et al., 2013) developed a 28-item self-report measure to assess the ability to mentalize, the Mentalization Scale (MentS), which assesses the general RF as well as the three distinct dimensions (i.e., Self-Oriented RF, Other-Oriented RF, and Motivation for RF). Although it has good psychometric evidence and a complex convergent and divergent validity, it lacks a convergent validity with the RFS. Fonagy et al. (2016) focused with the RFQ on a specific facet of RF, namely, the certainty and uncertainty of mental states. However, mentalizing is known as an umbrella concept consisting of distinctive dimensions (Luyten et al., 2019). The RFQ cannot claim to represent a holistic operationalization for the RF. Müller et al. (2013) highlighted that most items of the RFQ had a strong focus on self-orientated mentalizing and were more related to understanding the own behavior of oneself than feelings, wishes, or intentions. Both the RFQ and the MentS focus on the partial aspects of mentalizing but on different dimensions. The MentS assesses self- and other-oriented mentalizing as well as the motivation for mentalizing. Thus, although both questionnaires operationalize mentalizing, they could detect different aspects of it.

Furthermore, Mueller et al. (Luyten et al., 2019) questioned the methodological procedure of the RFQ scoring and tested the RFQ within clinical and nonclinical samples. They raised doubts about the validity of the RFQ and demonstrated by structure analyses that the RFQ seems unidimensional and lacked divergent validity between the dimension certainty (hypermentalizing) and clinical variables. Assessing mentalizing *via* self-report is a challenging task. Therefore, the MentS could be a promising alternative or addition to the RFQ within the mentalizing research.

## Hypotheses

This study aims to test the construct validity of the MentS questionnaire. For this purpose, the RFS is used. A correlation between the RFS values and the values of the MentS allows conclusions to be drawn about its convergent validity. We expect moderate to high correlations (0.5–0.9) between the dimensions of the individual and the global score of the MentS and the RFS. Another aim of this study is to replicate the internal consistency of Dimitrijević et al. (2018). We expect similar values as they obtained in the clinical group ranging from 0.60 to 0.79.

## Materials and Methods

### Participants and Procedure

Participants were 26 inpatients of a psychiatric hospital in Vienna (AT). Eligible participants were adults aged above 16 years with a clinical diagnosis of schizophrenia, schizoaffective disorder, delusional disorder, unipolar/bipolar depression, anxiety disorder, or cluster B personality disorder. Exclusion criteria were intellectual disability, acute psychotic episodes that required involuntary treatment according to legal act, patients who were not fluent in German, severe substance addiction, or neurological limitations. The psychiatric staff selected suitable patients. Participation in the study was voluntary. The participants were provided with written information and consented only after receiving a complete description of the study. They were given questionnaires and were interviewed by trained advanced medical students with a brief interview for RF. The interviews had been transcribed and had been coded by two reliable coders with the RFS.

### Measures

#### Brief Reflective Function Interview (BRFI)

The BRFI was published by Rudden, Milrod, and Target (Rudden et al., 2005) and was designed to assess the RF. It is a semi-structured interview consisting of 10 questions focusing on attachment-related contexts. It was developed as an alternative to the adult attachment interview (AAI; Main et al., 1985), which is, due to its complexity, hard to integrate into bigger sample sizes. The BRFI got validated by the AAI with good correlations (*r* = 0.71) as well as interrater correlations (ICC = 0.79). Although the AAI can assess RF and attachment representations, the BRFI can only be used to assess RF due to its focus on reflecting attachment figures and leaving out biographical episodes. The interviews get recorded and transcribed, afterwards to be analyzed with the RFS (Fonagy et al., 1998).

#### The RFS

The RFS was developed by Fonagy et al. (1998), and it allows to assess the capacity to mentalize dimensionally and categorically. The RFS measures the attachment-related mentalizing by determining to which extent the interviewee can give attachment relationships mental interpretations. Based on the attachment interviews, which get transcribed, an 11-point Likert scale, ranging from −1 (negative RF) to 9 (remarkable RF), is used for coding. The RFS has good psychometric properties (Taubner et al., 2013). After training for RFS, the interrater reliability ranges from 0.71 to 0.91 (Fonagy et al., 1996; Bouchard et al., 2008; Taubner et al., 2013).

#### The MentS

The MentS is a new self-reporting questionnaire of mentalization developed by Dimitrijević et al. (2018). It contains 28 items assessing the ability to mentalize by a whole scale as well as by three underlining dimensions, i.e., Self-Related Mentalization (MentS-S), Other-Related Mentalization (MentS-O), and Motivation to Mentalize (MentS-M). The psychometric properties were tested within clinical and nonclinical samples. Internal consistency was good for the non-clinical sample (α = 0.84) and acceptable for the clinical sample (α = 0.75). The subscales showed acceptable reliability for the non-clinical sample (α = 0.74–0.79) but lower reliability for the clinical sample (α = 0.60). The validity got tested by correlations with related constructs like attachment (*r* = −0.22–0.52), emotional intelligence (*r* = 0.22–0.67), and empathy (*r* = 0.35–0.51).

### Statistical Analysis

SPSS 21 was used for statistical analysis. The Shapiro–Wilk test was used to test for the normally distributed data. Due to the normally distributed data, Pearson's correlation and partial correlation were used to test for the correlation between MentS and RF. The Mann–Whitney *U*-test and the Kruskal–Wallis test were used for testing differences between demographics and RFS and MentS. Cronbach's alpha was calculated to test for internal consistency. The significance level was set to *p* < 0.05 (two-tailed) for all analyses.

## Results

### Sample Characteristics

The patients of our sample were aged between 18 and 74 years (*M* = 45.3, *SD* = 15.88), in which 53.8% were female. Most patients had an ICD-10 diagnosis of a psychosis spectrum disorder (i.e., schizophrenia: 30.8%, acute transient psychotic disorder: 15.4%, and schizoaffective disorder: 19.2%). Other diagnoses were bipolar affective disorder (11.5%), recurrent depressive disorder (19.2%), and borderline personality disorder (3.8%). There were no significant differences between sex and the RFS (Mann–Whitney *U* = 67.0, *n*_1_ = 14, *n*_2_ = 12, and *p* = 0.403) as well as MentS whole scale (Mann–Whitney *U* = 59.5, *n*_1_ = 14, *n*_2_ = 12, and *p* = 0.212) and subscales MentS-S (Mann–Whitney *U* = 76.5, *n*_1_ = 14, *n*_2_ = 12, and *p* = 0.705), MentS-O (Mann–Whitney *U* = 56.05, *n*_1_ = 14, *n*_2_ = 12, and *p* = 0.160), MentS-M (Mann–Whitney *U* = 74.0, *n*_1_ = 14, *n*_2_ = 12, and *p* = 0.631). Regarding education, there were no significant differences concerning RFS (Kruskal–Wallis test χ^2^ = 5.585 and *p* = 0.349), MentS whole scale (Kruskal–Wallis test χ^2^ = 7.379 and *p* = 0.194), MentS-Self (Kruskal–Wallis test χ^2^ = 5.062 and *p* = 0.408), MentS-Other (Kruskal–Wallis test χ^2^ = 3.373 and *p* = 0.643), and MentS-Motivation (Kruskal–Wallis test χ^2^ = 6.734, *p* = 0.241). Pearson's correlation between age and RFS showed significant moderate negative correlations (*r* = −0.465 and *p* = 0.017), and therefore, partial correlation had been used to control for age (see Table 1). The patients of our sample scored with a mean of 2.23 (*SD* = 2.03) regarding the RFS (see Table 2).

**Table 1:** Demographics and diagnoses (*N* = 26).

**Demographics**	***N* = 26**
**Gender**, ***n*** **(%)**	
Male	12 (46.2)
Female	14 (53.8)
Age, mean (SD; range)	45.31 (15.88; 18-74)
**Education**, ***n*** **(%)**	
Secondary school	6 (23,1)
High school	7 (26.9)
Apprenticeship	8 (30.8)
University	1 (3.8)
Other	3 (11.5)
**Diagnosis**, ***n*** **(%)**	
F20: Schizophrenia	8 (30.8)
F23: Transient psychotic disorders	4 (15.4)
F25: Schizoaffective disorders	5 (19.2)
F31: Bipolar affective disorder	3 (11.5)
F33: Recurrent depressive disorder	5 (19.2)
F60.3: Borderline PD	1 (3.8)

**Table 2:** Clinical data of the sample (*N* = 26).

	***M***	***SD***	**Range**	**Skew**	**Kurtosis**	**Shapiro-Wilk test**		**α**
						**Statistic**	***p***	
**MentS**								
MentS-S	22.23	6.08	10–34	0.023	−0.320	0.983	0.934^*^	0.687
MentS-O	33.88	7.10	23–50	0.783	−0.014	0.932	0.085^*^	0.796
MentS-M	34.31	7.37	17–50	−0.120	0.023	0.977	0.904^*^	0.556
MentS total	90.42	15.0	61–122	0.309	−0.237	0.982	0.811^*^	0.658
RFS	2.23	2.03	−1.00–6.50	0.327	−0.291	0.955	0.301^*^	

* *p < 0.05*.

### Association of MentS and MentS Subscales With the RFS

Significantly moderate to high positive correlations were obtained between MentS and RFS. Highest positive correlation was achieved with MentS whole scale (0.652, *p* = 0.000). Regarding the MentS subscales, MentS-O had the highest positive correlation (0.557, *p* = 0.004). MentS-S and MentS-M had similar moderate positive correlations (MentS-S 0.440, *p* = 0.028; MentS-M 0.413, *p* = 0.040) (see Table 3).

**Table 3:** Pearson's and partial correlations between RFS and MentS (*N* = 26).

	**1**	**2**	**3**	**4**	**5**
1. MentS-Self	1.00				
2. MentS-Others	0.375	1.00			
3. MentS-Motivation	0.062	0.434^*^	1.00		
4. MentS-Total	0.613^**^	0.839^**^	0.722^**^	1.00	
5. RFS	0.440^*^	0.557^**^	0.413^*^	0.652^**^	1.00

* *p < 0.05*,

** *p < 0.01*.

### Internal Consistency of the MentS

The Cronbach's alpha value obtained for the MentS whole scale was 0.617, which corresponds to questionable to acceptable effect (Blanz, 2015). Concerning the MentS subscales, the MentS-O achieved the highest score with 0.695, which indicates an acceptable internal consistency; the MentS-S reached a score of 0.687 (questionable to acceptable effect); and the MentS-M achieved the lowest score with 0.556 (poor).

## Discussion

The main goal of this study is to examine the construct validity of the MentS with the RFS within a sample of 26 severely ill inpatients at a psychiatric hospital. Furthermore, it was of interest to replicate the internal consistency of the MentS.

Concerning the primary goal of this study, the validity was satisfactory for the MentS whole scale according to our hypothesis with a large positive correlation with the RFS. Considering that the RFS and MentS are two different types of measurement (performance vs. self-report) and therefore could detect the various aspects of mentalizing (Fonagy et al., 2016), the correlations are quite high. Otherwise, the correlations between the RFS and MentS indicate that mentalizing could be, to some extent, deducible by self-assessment. Thus, patients effectively have a conscious idea of their skills to mentalize. Therefore, the MentS could be a promising alternative or addition to the RFS. The MentS subscales were slightly lesser but still highly correlated with the RFS. Since the RFS only examines the general RF, the MentS could help detect distinct RF dimensions like the self-/other-oriented RF or the motivation for RF. This finding could lead to a step forward in assessing the different underlying dimensions of RF, as Luyten et al. (2019) demanded.

The validity of a measurement is dependent on its reliability; therefore, the internal consistency was examined. In summary, the Cronbach's alpha for MentS, except for MentS-M, was close to the traditional cut-off of 0.70. Similar to the findings by Dimitrijević et al. (2018), the Cronbach's alpha value was lowest for MentS-M, whereas the other subscales and the whole scale were performed in relation to their sample akin. In comparison to their results, our sample obtained an overall lower score on Cronbach's alpha. Taking into account the sample characteristics, the lower values seem plausible due to more severe psychopathology. Dimitrijević et al. (2018) included patients with a borderline personality disorder who were inpatients as well as outpatients. In contrast, our sample consisted of inpatients at a psychiatric clinic (an acute psychiatric ward) with a high percentage of psychotic disorders at the beginning of treatment and a medication change phase.

The RF in our sample was low as expected, measured with both RFS and MentS. Interestingly, our sample scored, compared to the non-clinical and clinical samples of Dimitrijević et al. (2018), significantly lower on the MentS whole scale and subscales, except for the MentS-Motivation subscale. This is in line with theory and research that psychotic patients show severe mentalizing deficits (Richter et al., 2020). Furthermore, with the MentS, it seems possible to distinguish the capacity to mentalize between different disorders, which fosters the validity more. When aiming at diagnostic procedures that should have a prognostic or even predictive value, the operationalization of more detailed parameters—than that in the ICD/DSM-system—is appreciated. The transdiagnostic approach in the current precision medicine/psychotherapy demands such an approach.

In this context, the high negative correlation between RFS and age is noticeable. However, the percentage of psychotic disorders is relatively high in our sample. Thus, cognitive deficit analysis might somewhat explain this connection. Although the progressive deterioration of cognitive functioning in patients with psychosis is controversially discussed (McCleery and Nuechterlein, 2019), Thompson et al. (2013) found that older schizophrenic patients can be classified into subgroups of whom 40% exhibit modestly declining course and 10% more rapidly declining course. Our sample was relatively old, with a mean age of 45.3 years. Therefore, it could show a higher percentage of patients with progressive deterioration of cognitive functioning and side effects due to neuroleptic medication, which could lead to worse performance on the RFS. Interestingly, there were no significant correlations between MentS and age, which could indicate that a self-report of mentalizing is less sensitive to demographic variables, indicating that the MentS is less susceptible to demographic variables.

The MentS-O had the strongest correlation of the subscales with the RFS with 0.557. The MentS-S correlated quite less with the RFS with 0.440, although both show similar internal consistency. This could indicate that RFS and MentS-O assess similar aspects of mentalizing and that the RFS is, therefore, less suitable for evaluating the dimension of self-orientated mentalizing. Another consideration that should be taken into account is that psychotic patients show severe self-monitoring deficits and tend to externalize (Brookwell et al., 2013; Li et al., 2018) and that the MentS can therefore detect these deficits. From a psychodynamic point of view, these differences in self-and other-related mentalizing mirror the strong object dependency of the patient. Therefore, their severe fragmentation and tendency to externalize inner parts lead them to regulate their affects *via* projective identification. To perceive these externalizing mechanisms is of huge importance for treatment (Löffler-Stastka et al., 2010) and has a prognostic value (Löffler-Stastka et al., 2008). The psychiatrist/psychotherapist needs to contain them, work them through, and interpret them (Datz et al., 2019).

In a way, the RFQ assesses categories of the disturbance of the functions the MentS explores. Development of (secure) attachment (and a reflected view of memories of oneself related to others) is an aftermath of successful mentalization of mental states of oneself (MentS-S) and other (MentS-O). It is impossible without the motivation (MentS-M) to do so.

To interpret and deal with the external and internal reality, conceptions and borders of oneself and the other must first be well-developed and differentiated. Confrontation with reality and other individuals leads to the motivation to explore the interactions, attributing meaning to them and possibly intriguing their intents. But most importantly, this confrontation leads to acknowledging the existence of the other as separate from oneself and of the own limitations (e.g., to predict the mind of others and be understood). This is acquired in interactions with the relevant others in early childhood, the child (and humans in general) being in close dependency on a suitable environment (Fonagy et al., 2018; Luyten et al., 2020).

Thus, the motivation to engage with the social environment can be interpreted as a sign of the libido—the wish to live. A good enough caregiver provides a safe enough space for the development of epistemic trust (Fonagy and Campbell, 2017), and the internalization of a benevolent counterpart within repeated interaction differentiates and develops toward a distinguished view of the other related to the self, and so does safe attachment (Bowlby, 2008).

In psychosis, for instance, representations of the self, the other, and the assumptions, emotions, and memories attributed to them are not clearly and concisely integrable. Therefore, the conception of the reality of individuals who suffer from psychoses misses a sufficient overlapping with the one usually ascribed to it by normal/neurotic persons. What is typically applied to a dream world and primary process leaks into everyday moments; thus, functioning well-adapted to the situation at hand becomes impossible—with obvious social and attachment difficulties. As behavior results from feeling, sensing, and the cognitive appraisal of reality, it often reveals inner uncertainties, especially when insupportable and inexplicable ambiguity is not tolerable. Thus, this results in projective identification and acting out (i.e., language, gesture, and actions).

The motivation to interact with others can be compromised due to various reasons. However, suppose inner destructive and persecutive states are projected because the integration in a complete picture of oneself and the other had failed—motivation to relate to others and thus also mentalizing is presumably low. Therefore, motivation, an easily perceptible factor, could be an indirect measure of more profound disturbances not deducible by the patient even if the motivation is not caused but a consequence of those disturbances. Furthermore, motivation to mentalize could be an indirect measure of the severity of the pathology of the patient. An (untrained) environment reacting to excessive fear of the patient of losing the other and his/her wish to destroy the other will exhibit anger, refusal, confusion, and debasement (Bruns, 2021). Such aversive reactions are not easy to be contained by untrained relatives. This reaction again is supposed to influence the ability of the patient and wish to mentalize. It even aggravates harmful and intolerable affective states in the patient. Thus, making mentalizing even more unlikely, the vicious circle perpetuates itself, also *via* transference processes. Therefore, memorizing such affectively loaded experiences will likely trigger insupportable affects and corresponding defense mechanisms (i.e., denial and projective identification).

However, especially the narration of such memories, as measured with the RFS, will often be compromised in psychotic states. Language in psychotic individuals can have several different functions, e.g., affect regulation, and is not only communication, again leading to measurable mentalizing failure.

### Limitations

The findings of this study are facing some limitations. When examining the validity of a questionnaire, its reliability is of great importance. In our sample, the internal consistency is slightly below 0.70, and therefore, the validity could be lower than our suggested finding. Our goal is to use the gold standard of assessing RF using the RFS. Instead of using the original measurement, the AAI, by which the RFS got validated, we used the BRFI, which has satisfactory psychometrics but so far was not validated on a clinical population. In this sample, mentalizing was very low in both applied measures, RFS and MentS. This finding raises the question, whether this would be similar in a less severely affected sample with a broader variance of mentalizing abilities.

Due to the mixed sample consisting mainly of patients with psychotic disorders, affective disorders, and personality disorders, our findings are limited to generalizations for specific conditions and more for a particular treatment setting (acute psychiatric ward). Future studies could focus on the replication of our findings within (a) a bigger sample, (b) a more homogenous or heterogeneous sample, and (c) samples with broader variance in the RFS and MentS. Nonetheless, the economic assessment of mentalizing could be greatly utilized in specific clinical contexts like, for instance, in specialized departments for patients at ultra-high risk for developing a psychosis (UHR patients). In a recent longitudinal study, Boldrini et al. (2020) could highlight the predictive value of RF (measured with the RFS) in a sample of UHR patients for developing psychosis. Prediction models, in that case, are of great importance for early interventions and for influencing the course of illness. As the MentS is sensitive to detect externalizing mechanisms in the MentS-O dimension, this strength must be mentioned and observed further. Last but not least, our sample is relatively small and is therefore lacking statistical power.

## Data Availability Statement

The raw data supporting the conclusions of this article will be made available by the authors, without undue reservation.

## Ethics Statement

The study involving human participants was reviewed and approved by the Ethics-Committee of the City of Vienna on Oct, 15th 2016: EK 16-152-VK. The patients/participants provided their written informed consent to participate in this study.

## Author Contributions

FR did the statistical analysis including data management and the analyzing of the attachment interviews, writing, and submitting the manuscript. DS was reviewing the paper, doing correction in language as well as changes in content. HL-S had been the organizer of the clinical survey, was doing preparation of the ethical approval, and was reviewing as well as adding content to the whole manuscript. All authors contributed to the article and approved the submitted version.

## Conflict of Interest

The authors declare that the research was conducted in the absence of any commercial or financial relationships that could be construed as a potential conflict of interest.

## Abbreviations

RF: Reflective functioning
RFS: The Reflective Functioning Scale
RFQ: the Reflective Functioning Questionnaire
MentS: the Mentalization Scale
BRFI: Brief Reflective Function Interview
AAI: the adult attachment interview
MentS-S: Self-Related Mentalization
MentS-O: Other-Related Mentalization
MentS-M: Motivation to Mentalize.
